# Effects of tube voltage, radiation dose and adaptive statistical iterative reconstruction strength level on the detection and characterization of pulmonary nodules in ultra-low-dose chest CT

**DOI:** 10.1186/s40644-024-00770-z

**Published:** 2024-09-15

**Authors:** Yue Yao, Xuan Su, Lei Deng, JingBin Zhang, Zengmiao Xu, Jianying Li, Xiaohui Li

**Affiliations:** 1 Department of Radiology, the second Affiliated Hospital, Xi’an Jiaotong University, Xi’an, China; 2 GE HealthCare, Beijing, China

**Keywords:** Iterative reconstruction, ULDCT, Pulmonary nodule, Detection

## Abstract

**Objective:**

To explore the effects of tube voltage, radiation dose and adaptive statistical iterative reconstruction (ASiR-V) strength level on the detection and characterization of pulmonary nodules by an artificial intelligence (AI) software in ultra-low-dose chest CT (ULDCT).

**Materials and methods:**

An anthropomorphic thorax phantom containing 12 spherical simulated nodules (Diameter: 12 mm, 10 mm, 8 mm, 5 mm; CT value: -800HU, -630HU, 100HU) was scanned with three ULDCT protocols: Dose-1 (70kVp:0.11mSv, 100kVp:0.10mSv), Dose-2 (70kVp:0.34mSv, 100kVp:0.32mSv), Dose-3 (70kVp:0.53mSv, 100kVp:0.51mSv). All scanning protocols were repeated five times. CT images were reconstructed using four different strength levels of ASiR-V (0%=FBP, 30%, 50%, 70%ASiR-V) with a slice thickness of 1.25 mm. The characteristics of the physical nodules were used as reference standards. All images were analyzed using a commercially available AI software to identify nodules for calculating nodule detection rate (DR) and to obtain their long diameter and short diameter, which were used to calculate the deformation coefficient (DC) and size measurement deviation percentage (SP) of nodules. DR, DC and SP of different imaging groups were statistically compared.

**Results:**

Image noise decreased with the increase of ASiR-V strength level, and the 70 kV images had lower noise under the same strength level (mean-value 70 kV: 40.14 ± 7.05 (dose 1), 27.55 ± 7.38 (dose 2), 23.88 ± 6.98 (dose 3); 100 kV: 42.36 ± 7.62 (dose 1); 30.78 ± 6.87 (dose 2); 26.49 ± 6.61 (dose 3)). Under the same dose level, there were no differences in DR between 70 kV and 100 kV (dose 1: 58.76% vs. 58.33%; dose 2: 73.33% vs. 70.83%; dose 3: 75.42% vs. 75.42%, all *p* > 0.05). The DR of GGNs increased significantly at dose 2 and higher (70 kV: 38.12% (dose 1), 60.63% (dose 2), 64.38% (dose 3); 100 kV: 37.50% (dose 1), 59.38% (dose 2), 66.25% (dose 3)). In general, the use of ASiR-V at higher strength levels (> 50%) and 100 kV provided better (lower) DC and SP.

**Conclusion:**

Detection rates are similar between 70 kV and 100 kV scans. The 70 kV images have better noise performance under the same ASiR-V level, while images of 100 kV and higher ASiR-V levels are better in preserving the nodule morphology (lower DC and SP); the dose levels above 0.33mSv provide high sensitivity for nodules detection, especially the simulated ground glass nodules.

**Supplementary Information:**

The online version contains supplementary material available at 10.1186/s40644-024-00770-z.

## Introduction

With the wide application of low-dose computed tomography (LDCT), lung cancer screening has become increasingly popular, and the detection rate of lung nodules has gradually increased [1]. Lung cancer screening shows that most people with lung nodules are asymptomatic, but some patients are still at risk of having lung cancer [2]. To date, treating all non-calcified pulmonary nodules as potentially malignant lesions has been an accepted standard of practice and requires close monitoring until stabilization is demonstrated within 2 years [3, 4]; therefore, repeated LDCT follow-up evaluation is necessary for uncertain suspicious nodules to monitor diameter changes. The results of the National Lung Screening Trial (NLST) show that although the effective dose in LDCT averages about 1.5mSv each time for participants, the total effective dose could add up to about 8mSv over 3 years of screening [5], and the long-term radiation exposure from screening using current lung cancer screening protocols independently increases the risk of lung cancer other than smoking [6]. Therefore, finding appropriate ultra-low dose computed tomography (ULDCT) protocols for nodule screening and regular follow-up has become the focus of research.

In addition to physical protection, optimizing scanning protocol is a very effective way to reduce radiation exposure [7]. A number of strategies, such as the use of lower tube voltage and tube current automatic exposure control, selective in-plane shielding for reducing patient exposure and the use of iterative reconstruction (IR) algorithms to reduce image noise under low dose conditions have been developed [8–12]. Reducing the tube potential and tube current alone may impair image quality and reduce the diagnostic accuracy. However, at the same radiation exposure level, iterative reconstructions can significantly improve image quality and reduce noise, particularly through the utilization of the improved adaptive statistical iterative reconstruction -Veo (ASiR-V) algorithm [11, 13–15]. The wide applicability of ASiR-V enables ULDCT, whose effective dose can be comparable to that of chest radiography (< 0.2mSv) [16].

The nodule morphology is one of the strongest predictors of lung cancer in several retrospective analyses of NLST populations, and therefore accurately defining nodule morphology is an option to systematically improve screening efficiency [17, 18]. The study by Ye K, et al. [19] showed that ultra-low dose CT could be used for the detection of pulmonary nodules and studied the effect of different ASiR-V strength levels on their detection rate [20]. However, to the best of our knowledge, there is no study evaluating the combination of different tube voltages with ASiR-V strength levels on the detection of pulmonary nodules and the description of their morphology. Therefore, the purpose of this study was to evaluate the influence of different tube voltages combined with ASiR-V of different strength levels on the detection and morphology description of pulmonary nodules under ULDCT conditions. The detection and morphology description of pulmonary nodules was obtained by using a commercially available artificial intelligence software.

**Fig. 1:**
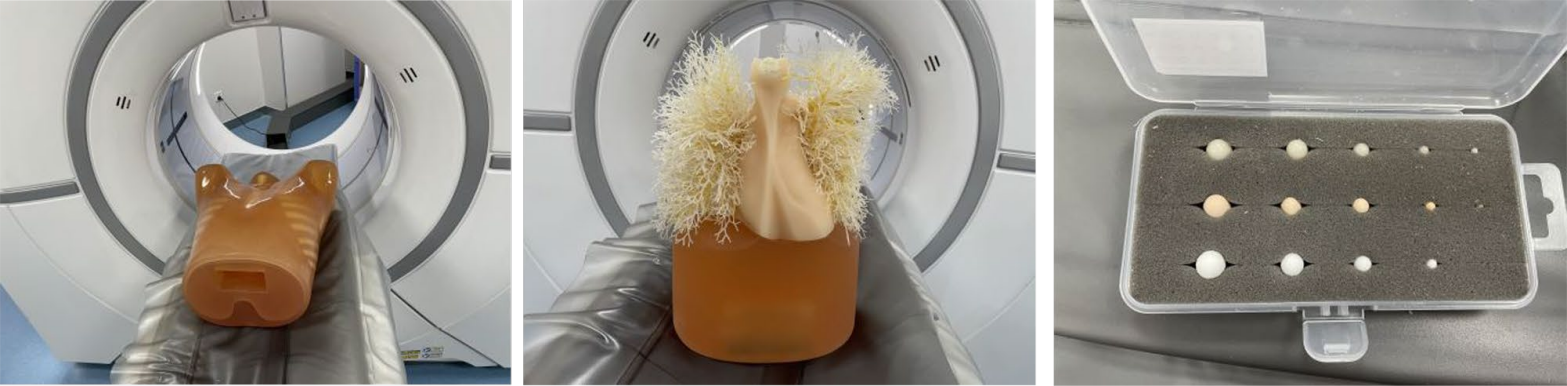
The Phantom and pulmonary nodules (ph-1, Kyoto Kagaku Inc, Japan). Trachea and pulmonary vessels were simulated by a mesh structure connected to the mediastinum. spherical nodules: diameters (5, 8, 10, 12 mm) and attenuations (−800, −630, +100 HU)

## Materials and methods

### Phantom

A thorax anthropomorphic phantom (Lungman ph-1, Kyoto Kagaku Inc, Japan) was used in our study (Fig. 1). The tracheal and pulmonary vessels were simulated by a mesh structure connected to the mediastinum. The lungs were simulated by the air naturally filled in the phantom. A total of 12 isolated spherical nodules including ground glass nodules (GGNs, -630HU and − 800HU) and solid nodules (SNs, 100HU) with different diameters (5, 8, 10, 12 mm) and different attenuations (− 800, − 630, +100 HU for each diameter) were randomly placed in the chest (Such as thoracic entrance, paratracheal, chest wall, etc.).

### CT protocols and image reconstruction

The phantom was scanned on a 256-row CT scanner (Revolution CT, GE HealthCare) with a standard low dose CT protocol and ULDCT protocols at three dose levels (Table 1). To achieve statistical robustness with the utilized image quality Metrics and provide the required number of images, each scan protocol was repeated 5 times without moving the phantom between acquisitions.

The raw data of each of the above different scans were reconstructed using filtered back projection (FBP) and ASiR-V with different strength levels of 30% (30%ASiR-V), 50% (50%ASiR-V) and 70% (70%ASiR-V). The images were reconstructed at 1.25-mm slice thickness and interval. The detection and size measurement of the nodules used lung window on the AI software. The SD values were measured manually on the images reconstructed with a standard kernel.

**Table 1:** ULDCT protocols at three dose levels and a standard low dose CT

		Dose 1 0.10-0.11mSv	Dose 2 0.32-0.34mSv	Dose 3 0.51-0.53mSv	Low Dose 1.28mSv
kVp/mA	70 kV	30 mA	100 mA	150 mA	
	100 kV	10 mA	30 mA	50 mA	120 mA
Rotation speed		0.5s			
Pitch		0.992:1			
Detector width		40 mm			
Reconstruction matrix		512 × 512			
DFOV		360 mm			
Reconstruction Algorithm		FBP, 30%ASiR-V, 50%ASiR-V, 70%ASiR-V			
Slice thickness and interval		1.25 mm			
Reconstruction kernel		lung and standard			

### CT data acquisition

All reconstructed images were then transferred to an image processing workstation equipped with an artificial intelligence (AI) software (Intelligent 4D Imaging System for Chest CT 5.5, YITU Healthcare) for image analysis and processing. The AI used in our study was a standalone commercially available software package that had not been learned or trained during the nodule detection in our study.

The AI software independently performed the assessment of pulmonary nodules in each image group. The type and long/short diameters of each nodule were recorded. The detection rate (DR) and deformation coefficient (DC) of pulmonary nodules of different groups were calculated for analyses. DC was defined as $\:\text{D}\text{C}=100\times\:(\text{L}\text{o}\text{n}\text{g}\:\text{d}\text{i}\text{a}\text{m}\text{e}\text{t}\text{e}\text{r}/\text{S}\text{h}\text{o}\text{r}\text{t}\:\text{d}\text{i}\text{a}\text{m}\text{e}\text{t}\text{e}\text{r}-1)$. Bias was quantified using size measurement deviation percentage (SP) defined as: $\:\text{S}\text{P}=100\times\:\frac{({\text{D}}_{\text{m}\text{e}\text{a}\text{s}\text{u}\text{r}\text{e}\text{d}}-{\text{D}}_{\text{t}\text{r}\text{u}\text{e}})}{{\text{D}}_{\text{t}\text{r}\text{u}\text{e}}}$, where D_measured_ was the mean diameter for a nodule in each image group, D_true_ was the true diameter of the physical nodule. SP and DC for each nodule size were calculated from measured diameters. The noise level for the image was the average value of the SD values of the pectoralis major, subscapularis, and erector spinae, and manually contouring and keeping the region-of-interest (ROI) in the same size as muscle tissue as possible.

### Statistical analysis

All statistical analyses were performed with SPSS statistical software (version 22.0, IBM SPSS Statistics). A two-sided p-value of < 0.05 was considered statistically significant. The SP and DC data of pulmonary nodules were compared using the non-parametric analysis of variance (Kruskal-Wallis test). The DR values for pulmonary nodules were calculated on the per-group basis using the number of the simulated nodules in the phantom and were compared by using the Chi-Square test. The nodule characteristics were also analyzed using the true properties of the simulated nodules. The sensitivity of ULDCT for detection of pulmonary nodules were calculated using the placed nodules as the reference.

## Results

### Image noise

At any given dose level and under the same ASiR-V strength level, there were no significant differences in image noise between the 70 kV and 100 kV scans, even though in general, the 70 kV images had slightly lower image noise; and noise gradually decreased with the increase of ASiR-V strength level ( Table 2).

**Table 2:** Image noise in different imaging groups

SD value	Dose 1		Dose 2		Dose 3	
	70 kV	100 kV	70 kV	100 kV	70 kV	100 kV
FBP	47.98 ± 7.75	50.12 ± 8.75	36.78 ± 4.47	40.57 ± 1.99	31.99 ± 4.60	35.88 ± 1.71
30%ASiR-V	40.72 ± 5.00	45.12 ± 2.87	29.27 ± 3.92	32.53 ± 1.72	25.32 ± 4.37	28.02 ± 1.76
50%ASiR-V	37.59 ± 5.25	39.31 ± 2.68	24.35 ± 3.61	27.37 ± 1.61	21.05 ± 4.23	22.92 ± 1.98
70%ASiR-V	34.25 ± 2.41	34.88 ± 5.23	19.80 ± 3.34	22.65 ± 1.61	17.16 ± 4.02	19.15 ± 2.75
mean-value	40.14 ± 7.05	42.36 ± 7.62	27.55 ± 7.38	30.78 ± 6.87	23.88 ± 6.98	26.49 ± 6.61

### Detection of pulmonary nodules

#### Comparison of nodule detection accuracy between 70 kV and 100 kV scan voltages at different dose levels

**Table 3:** Nodule detection analysis at different dose levels between 70 kV and 100 kV

	Dose 1		Dose 2		Dose 3		
	70 kV	100 kV	70 kV	100 kV	70 kV	100 kV	Low-Dose CT
True positive	141	140	176	170	181	181	192
True negative	99	100	64	70	59	59	48
Sensitivity	58.76%	58.33%	73.33%	70.83%	75.42%	75.42%	80.00%
*p*	> 0.05		> 0.05		> 0.05		

PS: The physical nodules were used as the reference standards. The total number of possible nodules was 240 under each scan protocol

For the LDCT scan and reconstruction, there were 240 possible nodules (12 nodules per phantom x 5 repeated scans x 4 reconstructions each scan), and 192 nodules were detected by the AI software, resulted in a DR of 80.00%. For the ULDCT protocols, there were 1440 possible nodules (12 nodules per phantom x 5 repeated scans x 2 kVs x 3 dose levels x 4 reconstructions each scan), and overall, 989 nodules were detected for a DR of 68.68%. Overall, 498 nodules were detected at 70 kV (498/720 for a DR of 69.16%); and 491 nodules were detected at 100 kV (491/720 for a DR of 68.19%), regardless of IR strength. An overview of the nodules found on LDCT and ULDCT is presented in Table 3. There were no significant differences in the detection (similar sensitivity) of nodules between 70 kV and 100 kV at all three radiation dose levels (all *p* > 0.05).

#### Comparison of the detection rates of different types of nodules

At any given dose level, the detection rates for GGNs and SNs nodules were independent of the ASiR-V strength level and tube voltage (all *p* > 0.05). When only focusing on GGNs, we found that the 70 kV/50%ASiR-V, 100 kV/70%ASiR-V and 70 kV/70%ASiR-V images had the highest DR value under Dose-1 (42.50%), Dose-2 (70.00%) and Dose-3 (72.50%), respectively. The DR values of 70 kV on SNs were overall higher than 100 kV in all three dose levels. Per-group detection results are shown in Table 4.

**Table 4:** The detection rates of GGNs and SNs on different ASiR-V levels

GGNs(DR%)		30%ASiR-V	50%ASiR-V	70%ASiR-V	FBP	Overall
Dose 1	70	37.50	42.50	37.50	35.00	38.12
	100	37.50	37.50	37.50	37.50	37.50
Dose 2	70	57.50	65.00	65.00	55.00	60.63
	100	57.50	60.00	70.00	50.00	59.38
Dose 3	70	57.50	65.00	72.50	62.50	64.38
	100	62.50	65.00	67.50	70.00	66.25
**SNs (DR%)**						
Dose 1	70	100.00	100.00	100.00	100.00	100.00
	100	100.00	100.00	100.00	100.00	100.00
Dose 2	70	100.00	100.00	100.00	100.00	98.75
	100	95.00	90.00	90.00	100.00	93.75
Dose 3	70	100.00	100.00	90.00	100.00	97.50
	100	100.00	100.00	75.00	100.00	93.75

PS: GGNs: Ground glass nodules; SNs: Solid nodules. There were 40 GGNs and 20 SNs in each scan protocol with 5 repeats. The nodule itself was the reference standard

#### Detection of nodules of different sizes at 70 kV and 100 kV under three dose levels

For the Dose-2 protocol, there were significant differences in the detection of nodules with CT attenuation value of 100HU and 5 mm in diameter (*p* = 0.035) and attenuation value of -800 HU and 10 mm in diameter (*p* = 0.047) between 70 kV and 100 kV, but there were no significant differences in nodule detection among other conditions (*p* > 0.05). No nodule with CT attenuation value of -800HU and 5 mm in diameter was detected under all three dose levels, and no nodule with CT attenuation value − 630HU was detected under Dose-1. The nodule with CT attenuation of -800HU and 12 mm in diameter was also not detected under Dose-1. (Table 5)

**Table 5:** Detection of nodules of different sizes at different dose levels

DR (%)		5 mm		*P*	8 mm		*P*	10 mm		*P*	12 mm	
		70 kV	100 kV		70 kV	100 kV		70 kV	100 kV		70 kV	100 kV
Dose 1	100HU	100.00	100.00	> 0.05	95.00	100.00	> 0.05	100.00	100.00	> 0.05	100.00	100.00
	-630HU	0	0	> 0.05	100.00	100.00	> 0.05	90.00	100.00	> 0.05	100.00	100.00
	-800HU	0	0	> 0.05	10.00	0	> 0.05	5.00	0	> 0.05	0	0
		> 0.05			> 0.05			> 0.05			> 0.05	
Dose 2	100HU	100.00	80.00	**0.035**	100.00	100.00	> 0.05	100.00	100.00	> 0.05	95.00	95.00
	-630HU	100.00	90.00	> 0.05	100.00	100.00	> 0.05	90.00	100.00	> 0.05	100.00	100.00
	-800HU	0	0	> 0.05	45.00	55.00	> 0.05	50.00	20.00	**0.047**	5.00	10.00
		> 0.05			> 0.05			> 0.05			> 0.05	
Dose 3	100HU	90.00	75.00	> 0.05	100.00	100.00	> 0.05	100.00	100.00	> 0.05	100.00	100.00
	-630HU	100.00	100.00	> 0.05	100.00	100.00	> 0.05	100.00	100.00	> 0.05	100.00	100.00
	-800HU	0	0	> 0.05	60.00	85.00	> 0.05	55.00	35.00	> 0.05	0	10.00
		> 0.05			> 0.05			> 0.05			> 0.05	

Ps: The number of nodules with different sizes and HU values in each group was 20

### Characterization of pulmonary nodules

#### Overall comparison of DC and SP of nodules

**Fig. 2:**
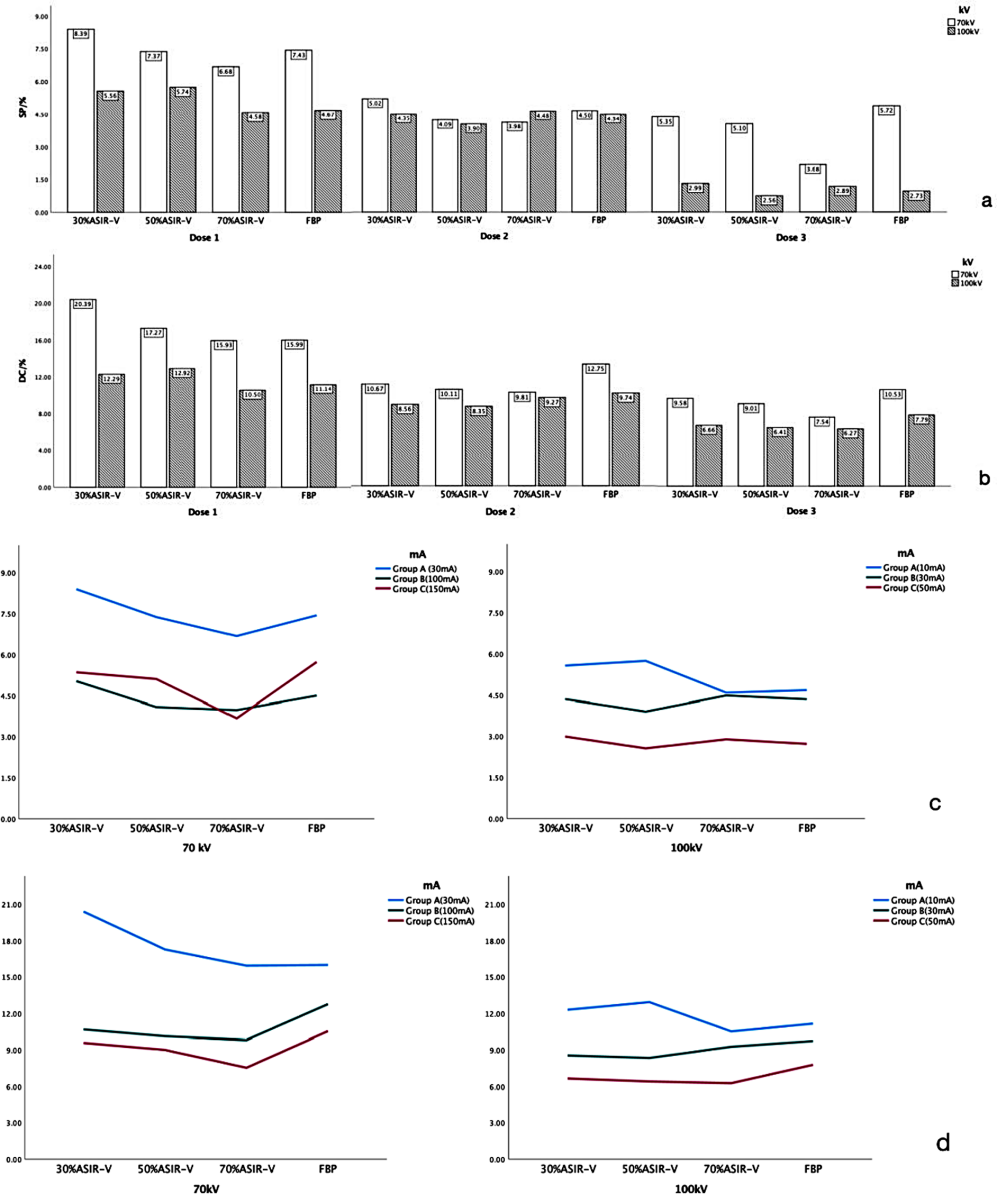
**a**) Histograms of the size measurement deviation percentage (SP) under different reconstruction methods; **b**) Histograms of the deformation coefficient (DC) under different reconstruction methods; **c**) Graphs of size measurement deviation percentage (SP) under different dose conditions (70 kV group on the left and 100 kV group on the right); **d**) Graphs of the deformation coefficient (DC) under different dose conditions (70 kV group on the left and 100 kV group on the right)

Regardless of the nodule type, there were no significant differences in SP and DC of nodules at similar radiation dose levels (70 kV/30 mA vs. 100 kV/10 mA, 70 kV/100 mA vs. 100 kV /30 mA, and 70 kV/150 mA vs. 100 kV/50 mA; *p* > 0.05). Except for 70%ASiR-V of Dose 2, 100 kV general had better (lower) DC and SP than 70 kV ( Fig 2).

#### Comparison of DC and SP of GGNs

There were significant differences in DC of GGNs between 70 kV and 100 kV with 30%ASiR-V in Dose-1 and Dose-3 (*p* < 0.05; *P* = 0.008; *p* = 0.024). The SP values of GGNs were statistically different between 70 kV and 100 kV with 30% and 50%ASiR-V and FBP in Dose-3 and FBP in Dose-1 (*p* = 0.014, *p* = 0.023, *p* = 0.03, *p* = 0.03; respectively). In other cases, there were no statistical differences in DC and SP (*p* > 0.05). (Table 6)

**Table 6:** The DC and SP of GGNs under different conditions

GGNs		kV	30%ASiR-V		50%ASiR-V		70%ASiR-V		FBP		*P*		mean	
DC/%	Dose-1	70	24.53		20.09		18.65		20.25		> 0.05		20.85	
		100	14.79		15.85		12.14		13.89		> 0.05		14.16	
	*P*		**0.008**		> 0.05		> 0.05		> 0.05					
	Dose-2	70	12.27		11.33		11.09		16.69		> 0.05		12.86	
		100	9.64		9.65		10.49		11.85		> 0.05		10.40	
	*P*		> 0.05		> 0.05		> 0.05		> 0.05					
	Dose-3	70	11.86		10.84		9.40		13.83		> 0.05		11.67	
		100	7.49		7.50		7.36		9.56		> 0.05		7.98	
	*P*		**0.024**		> 0.05		> 0.05		> 0.05					
GGNs		kV	30%ASiR-V		50%ASiR-V		70%ASiR-V		FBP		*P*		mean	
SP/%	Dose-1	70	10.66		9.10		7.95		9.45		> 0.05		9.29	
		100	7.43		7.65		5.96		5.98		> 0.05		6.76	
	*P*		> 0.05		> 0.05		> 0.05		**0.030**					
	Dose-2	70	6.39		4.96		4.87		5.76		> 0.05		5.50	
		100	5.15		4.46		5.54		5.26		> 0.05		5.10	
	*P*		> 0.05		> 0.05		> 0.05		> 0.05					
	Dose-3	70	6.25		5.75		4.10		6.72		> 0.05		5.82	
		100	3.49		3.14		3.46		3.22		> 0.05		3.33	
	*P*		**0.014**		**0.023**		> 0.05		**0.03**					

PS: SP: size measurement deviation percentage; DC: deformation coefficient; GGNs: Ground glass nodules; SNs: Solid nodules

#### Comparison of DC and SP of SNs

The DC values of SNs between 70 kV and 100 kV under 30%, 50%, 70%ASiR-V at Dose-1 and 30%ASiR-V at Dose-3 were statistically different (*P* < 0.05). There were significant differences in SP of SNs between 70 kV and 100 kV under the conditions of 30%, 50%, 70%ASiR-V at Dose-1 and 50%ASiR-V at Dose-3 (*p* < 0.05); but no significant differences for the other conditions (*p* > 0.05). (Table 7)

**Table 7:** The DC and SP of SNs under different conditions

SNs		kV	30%ASiR-V	50%ASiR-V	70%ASiR-V	FBP	*P*	mean
DC/%	Dose-1	70	12.11	11.61	10.72	7.45	> 0.05	10.48
		100	7.40	7.04	7.21	5.64	> 0.05	6.8
	*P*		**0.010**	**0.008**	**0.032**	> 0.05		
	Dose-2	70	7.48	7.79	7.24	4.86	> 0.05	6.84
		100	6.38	5.59	6.55	5.64	> 0.05	6.03
	*P*		> 0.05	> 0.05	> 0.05	> 0.05		
	Dose-3	70	5.03	5.34	3.82	3.91	> 0.05	4.52
		100	4.99	4.23	4.08	4.23	> 0.05	4.38
	*P*		**0.001**	> 0.05	> 0.05	> 0.05		
SNs		**kV**	**30%ASiR-V**	**50%ASiR-V**	**70%ASiR-V**	**FBP**	*P*	**mean**
SP/%	Dose-1	70	3.85	3.89	4.11	3.38	> 0.05	3.81
		100	1.91	1.90	1.80	2.10	> 0.05	1.93
	*P*		**0.008**	**0.004**	**0.003**	> 0.05		
	Dose-2	70	2.28	2.43	2.18	1.97	> 0.05	2.21
		100	2.72	2.71	2.11	2.53	> 0.05	2.52
	*P*		> 0.05	> 0.05	> 0.05	> 0.05		
	Dose-3	70	3.52	3.79	2.81	3.71	> 0.05	3.48
		100	1.99	1.39	1.74	1.72	> 0.05	1.71
	*P*		> 0.05	**0.01**	> 0.05	**0.008**		

PS: SP: size measurement deviation percentage; DC: deformation coefficient; GGNs: Ground glass nodules; SNs: Solid nodules

The DC and SP of solid nodules were better (lower) than those of ground glass nodules, regardless of the scan condition. Especially under Dose-1, the differences between DC and SP of different nodule types were the largest.

## Discussion

The objective of this study was to investigate the effect of ASiR-V algorithm of different strength levels on the detection and characterization of pulmonary nodules in ULDCT at different dose levels. To further isolate the impact of tube voltage (kV), similar doses were generated at tube voltages of 70 kV and 100 kV through tube current modulation. Under the conditions of 0.51-0.53mSv in ULDCT, the combination of 70 kV and 70%ASiR-V generated the highest detection rate of 72.5% for GGNs, while the combination of 100 kV and high ASiR-V strength levels was better in preserving the forms of nodules.

In our study, the average effective doses of ULDCT were 0.105mSv, 0.33mSv, 0.52mSv, and the effective dose of the reference low dose CT was 1.28mSv. We found that the dose of ULDCT was reduced by 91.79%, 74.21%, 59.38%, respectively, but the sensitivity of nodule detection was only reduced by 27.08%, 9.89%, and 5.73%, respectively. Although the sensitivity for nodule detection was decreased, the majority (78%) of the undetected nodules had size less than 5 mm. It is well known that small nodules smaller than 5 mm have a very low risk of developing malignant tumors (less than 1%) [1, 21–23].

The overall DR of our ULDCT was 68.68%, with a maximum of 72.5% for GGNs (162 of which were not detected with a diameter of 5 mm). Botelho [24] et al. suggested that the minimum radiation dose to meet the diagnostic requirements for patients with a diameter of 5 mm should be 0.238 mSv when using fixed tube currents. Our study showed that PNs of 5 mm with an attenuation value of 100HU could be detected 100% (20/20) at 0.105mSv (Dose-1), but the detection ability of GGNs was limited at Dose-1 regardless of whether 70 kV or 100 kV was used. However, for nodules larger than 5 mm in Dose-1, CT attenuation values of 100HU and − 630HU could be detected at high detection rates (100HU: 70 kV:59/60, 100 kV:60/60; -630HU: 70 kV:58/60, 100 kV:60/60); and nodules with CT attenuation value of -800HU were mostly undetectable regardless of their sizes. At doses above 0.33mSv (Does-2 and Dose-3), there was essentially no change in the detection of nodules larger than 5 mm with CT attenuation values of 100HU and − 630 HU (100HU: 70 kV:119/120, 100 kV:119/120; -630HU: 70 kV:118/120; 100 kV:120/120). At the same times, the detection of 5 mm nodules with attenuation value of -800HU remained poor, but the detection for sizes larger than 5 mm increased significantly (70 kV:43/120; 100 kV:43/120). Considering that the minimum acceptable sensitivity of the screening test is 80% [25], ULDCT is not recommended for screening GGNs with CT attenuation of -800 HU or lower, and a higher radiation dose is recommended.

With regard to image quality, we found that when the dose was reduced, the image noise increased, the edge of the nodule was irregular, and the measurement error was prone to occur. The lower the radiation dose, the more serious the error and the larger the deformation index, and there was a significant difference in DC and SP between the 70 kV and 100 kV groups (*p* < 0.05). The effect of different dose levels on GGNs was stronger than SNs. At 70 kV, DC and SP decreased gradually with the increase of reconstruction strength. We found that the low kV and iterative reconstruction algorithm at high strength levels had the greatest effect in reducing DC and SP on the nodules with low CT attenuation values. Other studies have demonstrated that in ULDCT the use of iterative reconstruction algorithms, such as ASiR-V, and deep learning-based reconstruction algorithms could significantly reduce image noise and improve image quality [26–28]. The influence of different iteration strength levels on the image is mainly reflected in image quality, resolution, and noise level. Sui et al. [29] showed that there was no effect on the size measurement of nodules at low and ultra-low doses. However, our study found that there were deviations in the size measurement of nodules when combined with different ASiR-V levels. In this study, three representative strength levels of low, medium, high (30%, 50%, 70%) were used. however, we found that some features of the nodules could be distorted when iterative reconstruction algorithms with low strength levels were used, resulting in errors in diagnosis. Therefore, based on our results we recommend using 50% and 70% ASiR-V for image reconstruction to better preserve the nodule characteristics in ULDCT.

This study has several limitations: First, the sample size was small, only three kinds of CT attenuation value nodules were analyzed; Second, AI software from only one commercial company was used to obtain information. After the commercial AI software was obtained, the data training was not re-conducted. As far as we know, the general commercial AI software rarely performed ULDCT training, so the obtained information may not be 100% consistent with the training data, resulting in certain deviations in data analysis (for example, the nodules of -800HU failed to be detected, resulting in a low detection results). It is suggested that multiple software should be used for verification in the future. Third, this study was carried out on a phantom of the lung, which should be extended to real patient image analysis in the future.

In conclusion, the use of ULDCT combined with ASiR-V provides acceptable image quality at greatly reduced radiation exposure to patients, and there are no significant differences in the detection of nodules between 70 kV and 100 kV. At the same time, it is not recommended to choose too low dose conditions for finding GGNs. We recommend that dose levels above 0.33mSv be considered for screening, to ensure nodule detection and characteristic assessment. For patients with small nodules, 100 kV combined with higher ASiR-V strength levels (more than 50%) should be used to follow up the changes of nodules.

## Electronic supplementary material

Below is the link to the electronic supplementary material.

Supplementary Material 1

## Data Availability

No datasets were generated or analysed during the current study.

## Abbreviations

ASiR-V: Adaptive statistical iterative reconstruction -Veo
FBP: Filtered back projection
IR: Iterative reconstruction
AI: Artificial intelligence
LDCT: Low-dose chest CT
ULDCT: Ultra-low-dose chest CT
DR: Detection rate
DC: Deformation coefficient
SP: Deviation percentage
PN: Pulmonary Nodule
SNs: Solid nodules
GGNs: Ground glass nodules
